# Fatty Acid Synthase Inhibitor Platensimycin Intervenes the Development of Nonalcoholic Fatty Liver Disease in a Mouse Model

**DOI:** 10.3390/biomedicines10010005

**Published:** 2021-12-21

**Authors:** Meng Su, Danfeng Cao, Zhe Wang, Yanwen Duan, Yong Huang

**Affiliations:** 1 Xiangya International Academy of Translational Medicine, Central South University, Changsha 410013, China; msu2017@hnu.edu.cn (M.S.); 177501006@csu.edu.cn (D.C.); Wangz1525@csu.edu.cn (Z.W.); 2 Hunan Engineering Research Center of Combinatorial Biosynthesis and Natural Product Drug Discovery, Changsha 410011, China; 3 National Engineering Research Center of Combinatorial Biosynthesis for Drug Discovery, Changsha 410011, China

**Keywords:** non-alcoholic fatty liver diseases, platensimycin, de novo lipogenesis, FASN

## Abstract

Non-alcoholic fatty liver disease (NAFLD) is a chronic liver disease affecting about 25% of world population, while there are still no approved targeted therapies. Although platensimycin (PTM) was first discovered to be a broad-spectrum antibiotic, it was also effective against type II diabetes in animal models due to its ability to inhibit both bacterial and mammalian fatty acid synthases (FASN). Herein, we report the pharmacological effect and potential mode of action of PTM against NAFLD in a Western diet/CCI_4_-induced mouse model and a free fatty acids (FFAs)-induced HepG2 cell model. The proper dose of PTM and its liposome-based nano-formulations not only significantly attenuated the Western diet-induced weight gain and the levels of plasma total triglycerides and glucose, but reduced liver steatosis in mice according to histological analyses. Western blotting analysis showed a reduced protein level of FASN in the mouse liver, suggesting that PTM intervened in the development of NAFLD through FASN inhibition. PTM reduced both the protein and mRNA levels of FASN in FFAs-induced HepG2 cells, as well as the expression of several key proteins in lipogenesis, including sterol regulatory element binding protein-1, acetyl-CoA carboxylase, and stearoyl-CoA desaturase. The expression of lipid oxidation-related genes, including peroxisome proliferator activated receptor α and acyl-CoA oxidase 1, was significantly elevated. In conclusion, our study supports the reposition of PTM to intervene in NAFLD progression, since it could effectively inhibit de novo lipogenesis.

## 1. Introduction

Non-alcoholic fatty liver disease (NAFLD) is characterized by hepatic steatosis, which may further progress to non-alcoholic steatohepatitis (NASH) with hepatocyte damage and inflammation [1]. The NAFLD epidemic affects about 25% of the global population, with a significant increase in Asia [2,3]. Moreover, 40% of these patients are classified as non-obese and almost a fifth of them are lean, while they might develop liver cirrhosis more rapidly than obese individuals with NAFLD [4,5]. Many metabolic diseases including type II diabetes, obesity, hyperlipidemia, and hypertension are associated with NAFLD [6]. In particular, NAFLD is identified as an important risk factor for severe coronavirus disease 2019 (COVID-19) outcomes, probably due to the increased expression of angiotensin converting enzyme 2 and transmembrane protease serine 2 in multiple tissues of NAFLD patients infected with severe acute respiratory syndrome coronavirus 2 (SARS-CoV-2) [7,8].

**Figure 1:**
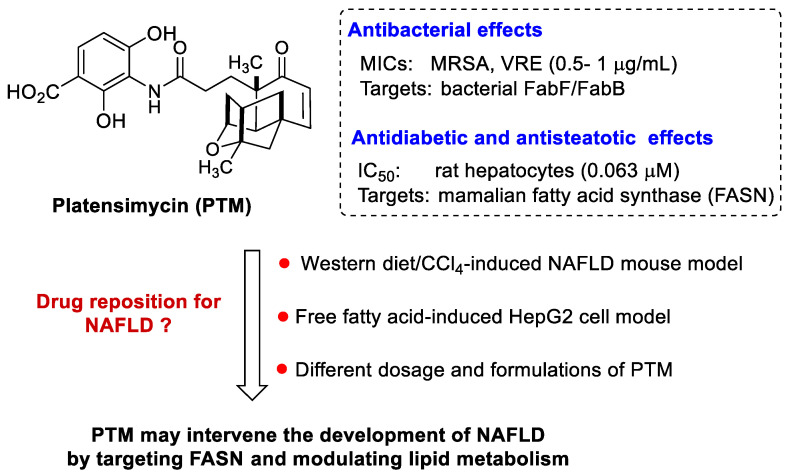
Targeting de novo lipogenesis against NAFLD by platensimycin.

Despite the high prevalence and rising incidence of NAFLD, there are no approved therapeutics in the markets. One major reason is that NAFLD is a multisystemic disease with successive liver abnormalities and extra-hepatic complications. The accumulation of toxic lipids in liver results in hepatocyte damage, followed by fibrogenesis and genomic instability, which eventually leads to cirrhosis and liver carcinoma. There are many on-going-phase II and III clinical trials against NASH to explore various intrahepatic targets, including hepatocyte injury, inflammation, and fibrosis. For example, dietary carbohydrates can be converted to fatty acids in hepatocytes via de novo lipogenesis (DNL) to produce triglycerides (TGs) [9]. This contributes to hepatic steatosis in individuals with NAFLD with 26% TGs in the liver from DNL, and a two-fold higher rate of DNL than that of individuals with low liver fat [10,11]. Therefore, lipogenic enzymes, such as acetyl Co-A carboxylase (ACC) and fatty acid synthase (FASN) in the DNL pathway, are ideal targets for NAFLD treatment (Figure 1).

A large number of ACC inhibitors, such as GS-0976, are highly effective in reducing hepatic steatosis, improving insulin sensitivity, and favorably affecting dyslipidemia in animal models of NAFLD and NASH [12,13,14,15,16,17,18]. However, ACC inhibition decreases malonyl-CoA levels that may affect the synthesis of polyunsaturated fatty acids and it would also increase circulating TGs [15,19]. FASN catalyzes the final step of DNL, converting acetyl-CoA and malonyl-CoA to palmitate. FASN gene expression level is elevated in NAFLD patients [20]. Recent clinical data using FASN inhibitors, such as FT-4101 and TVB-2640, have inhibited DNL and robustly improved hepatic steatosis without elevating circulating TGs [21,22,23].

Despite these great strides, there are no safe and effective FASN inhibitors available for the treatment of NAFLD in clinics. Platensimycin (PTM), a highly morphed and investigational diterpenoid isolated from soil *Streptomyces*, is a potent and highly selective inhibitor of mammalian fatty acid synthase (EC_50_ = 0.1 μM) (Figure 1) [24]. Although it was first discovered to be a potent bacterial FabF inhibitor [25], Singh and co-worker later discovered that it also inhibits fatty acid synthesis and hepatic de novo lipogenesis in rat primary hepatocytes, while it does inhibit sterol synthesis. PTM was effective against several type II diabetic animal models, including *db*/+ and *db*/*db* mice, diet-induced obese (eDIO) mice, and rhesus monkeys [24,26]. NAFLD and type II diabetes share many common risk factors, including insulin resistance. A bidirectional relationship between NASH and type 2 diabetes has recently been proposed, based on the potential beneficial effect of anti-diabetic and metabolic investigational drugs on NASH [27]. Therefore, we hypothesized that PTM might be effective against NAFLD, due to its capability to inhibit DNL in mice and monkey models of type 2 diabetes. In this study, we explored the preventive and therapeutical effects of PTM and its liposomal formulation using a NAFLD mouse model and a HepG2 cell model. PTM could not only effectively prevent the development of NAFLD in mice with a low dosage, but alleviate the severity of liver steatosis, by inhibiting the expression of *FASN* and related metabolic genes. Our study suggests that PTM could be used as a promising drug lead against NAFLD.

## 2. Materials and Methods

### 2.1. Reagents

PTM was obtained according to our previous report [28]. PTM-loaded liposome nanoparticles (Lip/PTM) and mannose-modified liposome nanoparticles (M-Lip/PTM) were prepared by the dehydration−rehydration method based on our previous method [29].

### 2.2. Cell Culture and Treatment 

Human hepatocyte cell line HepG2 was cultured in Dulbecco’s modified eagle medium (DMEM) (Thermo Fisher Scientific, Shanghai, China), supplemented with 10% fetal bovine serum (FBS) (Gemini), 100 units/mL of penicillin and 10 mg/mL of streptomycin (Thermo), and maintained under standard conditions of 37 °C with 5% CO_2_ in a humidified incubator (Thermo). In order to establish the NAFLD cellular model, oleic acid (OA) (Sangon, Shanghai, China) and palmitic acid (PA) (Sigma-Aldrich, Shanghai, China) at a ratio of 2:1 (0.67 and 0.33 mM, respectively) were mixed with fatty acid-free bovine serum albumin (BSA) (Bomei, Hefei, China) [30]. After attaining 80% confluence, HepG2 cells were cultured with FBS-free DMEM medium overnight and then exposed to 1 mM FFAs (OA/PA, 2:1) containing 1% BSA, with or without PTM for 48 h. Meanwhile, the control cells were treated with 1% BSA only. After treatment, cells were washed twice with phosphate buffered saline (PBS) and fixed in 10% paraformaldehyde for 30 min at room temperature. Next, the Oil red O staining working buffer (stock solution: 5 mg/mL in isopropanol, working solution: 60% Oil red O stock solution) was added and kept for 15 min, then washed with PBS and 60% isopropanol. After staining, cells were washed thoroughly with PBS (3×) and stained with hematoxylin dye solution. To quantify the Oil red O content, samples were extracted by isopropanol at room temperature for 5 min, and the optical density of each sample was read at 510 nm.

### 2.3. Cell Viability

Cell viability assays were performed using Cell Counting Kit-8 (CCK-8) (Dojindo Molecular Technologies, Shanghai, China) according to manufacture instructions. Briefly, HepG2 cells were plated at 3000 cells per well in 96-well plates in a medium containing 10% FBS. After treatment with various concentrations of PTM for another 48 h, CCK8 solution (10 μL) was added to each well and incubated for 1 h. Cell viability was finally measured using a microplate reader (TECAN) at a wavelength of 450 nm.

### 2.4. Animals

All animal studies were approved by Animal Ethics Committee of Central South University (CSU) and carried out at the Department of Laboratory Animals in CSU. Seven-week-old C57BL/6J male mice were purchased from Hunan Silaikejingda Experimental Animal Company Limited (Changsha, China). The mice had free access to chow and water. Animals were acclimatized for 5–6 days prior to initiating the study. 

### 2.5. Animal Study

The mouse model with NAFLD was similarly established as described [31]. In brief, C57BL/6J mice were fed with Western diet containing 42% fat, 44% sucrose, and 0.2% Cholesterol (*w*/*w*) (Trophic, TP26305) and a high solution of D-fructose and D -glucose (23.1 and 18.9 g/L). Meanwhile, CCl_4_ at the dose of 0.2 mL/kg of body weight was injected intra-peritoneally (IP injection) once per week. Mice in the normal group were fed with a normal chow diet (Trophic, TP 26358) and normal tap water, while corn oil was injected intra-peritoneally once per week. Mice were randomly distributed into nine groups as the following: Normal control group (normal diet/corn oil, *n* = 10); NAFLD group (WD/CCl4, *n* = 10); NAFLD + EGCG group (oral, 100 mg/kg for 13 weeks, *n* = 10); NAFLD + PTM group (oral, 10 or 50 mg/kg for 13 weeks, *n* = 10); NAFLD + PTM group (ip.10 mg/kg, *n* = 6 or oral 50 mg/kg for 5 weeks, *n* = 10); NAFLD + Lip/PTM group (IP injection, 10 mg/kg for 5 weeks, *n* = 6); NAFLD + M-Lip/PTM group (IP injection, 10 mg/kg for 5 weeks, *n* = 6).

### 2.6. Histopathology Analysis and Lipid Measurement

Fresh liver tissues were fixed in 10% formalin, processed into 6-μm-thick paraffin sections, and stained with hematoxylin and eosin (H&E). Liver tissues were stained with Oil red O staining for the assessment of hepatic steatosis according to the manufacturer’s instructions (American Master Tech, Lodi, CA, USA). Images (200×) were captured using a Leica microscope (Center Valley, PA, USA) and examined in a blinded manner. For the determination of the lipid spectrum, the TG and TC contents were measured with a biochemical analyzer (Leidu, Shenzhen, China). The TC and TG contents in the liver tissue were quantified with a kit (Jiancheng, Nanjing, China) according to manufacturer instructions. 

### 2.7. Determination of Plasma Alanine Aminotransferase Concentration

Alanine aminotransferase in serum was quantified using a commercial kit (Jiancheng bioengineering, Nanjing, China).

### 2.8. Oral Glucose Tolerance Test (OGTT)

Mice were fasted overnight and the baseline blood glucose levels were measured using the tail-vein blood. Five days before the termination of experiments, their blood glucose levels were determined using a glucose meter (Sinocare, Changsha, China) at different time points (0, 30, 60, 90, and 120 min after oral gavage of glucose (2 g/kg)). 

### 2.9. Quantitative Real-Time PCR

HepG2 cells were plated in 6-well tissue culture-treated plates in DMEM (2 × 10^5^ cells/per well). After 24 h, cells were supplemented with a mixture of free fatty acid (FFAs, OA:PA = 2:1) containing 1% BSA at a total final concentration of 1 mM, and treated with PTM or EGCG for 48 h. Next, the total RNA of treated cells was individually extracted using the TRIzol reagent (Takara), and ~1 μg of each RNA was reverse-transcribed to cDNA in a 20 μL reaction using PrimeScript™ RT reagent Kit with gDNA Eraser (Takara, Dalian, China). The respective gene expression levels were measured under the following conditions: 45 cycles of 95 °C for 10 s, 60 °C for 30 s, and 72 °C for 30 s using a LightCycler 96 (Roche) and quantified using the cycle threshold (Ct) value. The target genes were normalized relative to the reference gene encoding β-actin. 

### 2.10. Western Blot Analysis

After HepG2 cells were plated in 6-well tissue culture-treated plates (2 × 10^5^ cells/per well), the cells were supplemented with a mixture of FFAs (OA:PA = 2:1) at a final concentration of 1 mM. PTM or EGCG (10 or 50 μM) was used to treat HepG2 cells for 48 h. After the treated cells were washed with ice-cold PBS and lysed with RIPA lysis buffer (Beyotime, Shanghai, China), the cell supernatant was collected and protein concentrations were measured using the BCA Protein Assay Kit (Beyotime, Shanghai). An equal amount of protein (20 μg) was separated by 8% SDS-PAGE gel and the proteins were transferred to a polyvinylidene fluoride (PVDF) membrane (Millipore, Billerica). The membranes were blocked with 5% skimmed milk in tris-buffered saline tween (TBST) buffer for 1 h and then incubated with the following primary antibodies anti-FASN (rabbit, 273 KDa, ab128870, 1:30,000 dilution, Abcam) and GAPDH (rabbit, 36 KDa, AP0066, 1: 10,000 dillution, Bioworld) overnight at 4 °C. The resulting PVDF membrane was next washed in TBST three times and incubated with the secondary antibodies (anti-rabbit IgG, BS13278, 1:10,000 dilution, Bioworld) for 1 h at room temperature. Immunoreactive bands were visualized by the ChemiDoc XRS+ imaging system (Bio-Rad). Human glyceraldehyde-3-phosphate dehydrogenase (GAPDH) was used as an internal control. For the quantification of the protein bands, Image J (National Institutes of Health, Bethesda, MD, USA) was used to obtain the densitometric values.

### 2.11. Statistical Analysis

All data were represented as means ± S.E.M of three independent experiments. All statistical analyses were conducted using prism software using T test. *p* < 0.05 was considered to be statistically significant.

## 3. Results

### 3.1. The Protective Effects of PTM against the Development of NAFLD in a Murine Model Induced by Western Diet and CCl_4_

**Figure 2:**
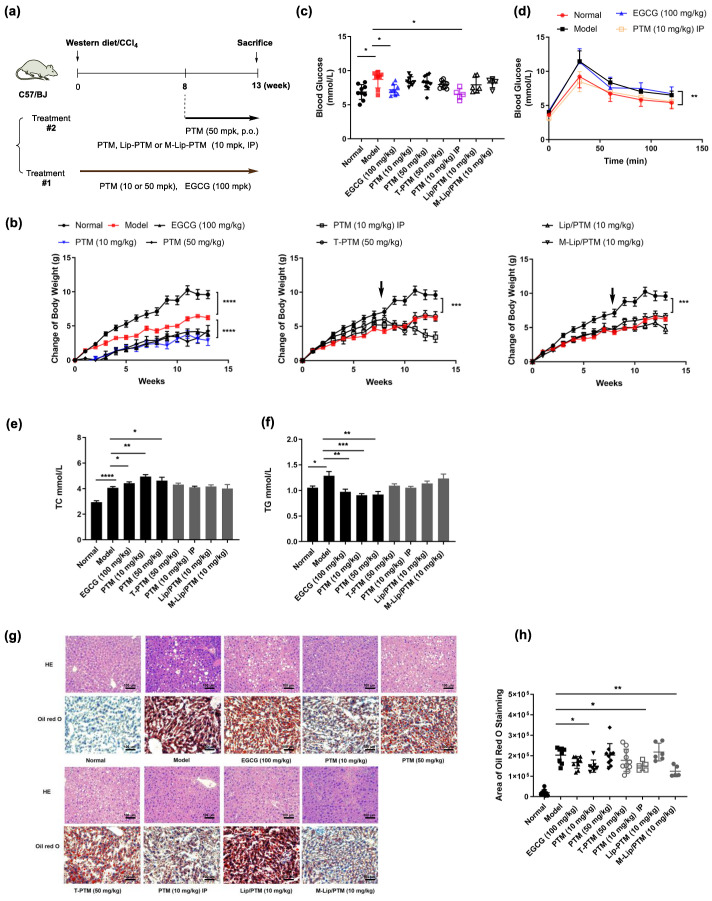
PTM reverses hepatic steatosis in mice induced by western diet and CCl_4_. (**a**) Schematic diagram of the experimental design for the in vivo experiments. #1: treatment for 13 weeks, #2: treatment for 5 weeks. (**b**) Body weight change. (**c**) Plasma cholesterol. (**d**) Plasma triglyceride. € Free diet blood glucose. (**f**) Oral glucose tolerance test. (**g**) Representative images of liver sections stained with H&E or Oil red O. (**h**) Quantification of Oil red O staining. Data are expressed as means ± SEMs, *n* = 5–10 mice per group. * *p* < 0.05, ** *p* < 0.01, *** *p* < 0.001, **** *p* < 0.0001.

Various murine models have been used to replicate the characteristics of human NAFLD, such as hepatocyte ballooning, liver steatosis, and lobular inflammation, in order to study their pathogenesis mechanisms and the development of transformative treatments. Tsuchida and co-workers recently developed a murine NASH model with rapid disease progression, by the combination of Western diet and intraperitoneal injection of CCl_4_ [31]. The WD/CCl_4_-treated C57BL/6J mice showed severe steatohepatitis and stage 3 bridging fibrosis as early as 12 weeks, which has been widely used to advance our understanding of NAFLD, fibrosis, and the progression to hepatocellular carcinoma in a shorter time window. Therefore, we first evaluated if PTM could prevent the development of NAFLD in this murine model, using (-)-epigallocatechin-3-gallate (EGCG) as a control (Figure 2a). EGCG is a plant polyphenol widely used as a dietary supplement, which has shown protective and therapeutical effects on NAFLD mice and small-scale clinical trials [32]. We have previously shown that the liposomal delivery of PTM may improve its pharmacokinetic properties by prolonging its half-life in the circulation system, since over 99% PTM may be secreted through kidney if free PTM was administered via oral or iv injection [33]. We therefore also evaluated the therapeutic effects of Lip/PTM and M-Lip/M, two liposomal formulations of PTM against NAFLD in this model.

The treatment of C57BL/6J mice by WD/CCl_4_ resulted in the decrease of mouse body weight in comparison with mice with chow diet and corn oil injection, which suggests that chronic injection of CCl_4_ (0.2 mg/kg) likely induced liver injury and caused the significant weight loss (Figure 2b and Figure S1). There was a significant increase of TGs in the plasma and liver of WD/CCl_4_-treated mice in comparison to the normal group (Figure 2c,d and Figure S2). In addition, both levels of the free diet blood glucose and the fasting blood glucose of WD/CCl_4_-treated mice increased, while their glucose tolerance declined (Figure 2e,f and Figure S3). H&E staining indicated that the liver of the WD/CCl_4_-treated group had marked accumulation of lipid droplets, which is consistent with the over two-fold more area stained by Oil red O than that of normal groups (Figure 2g,h). However, the level of serum alanine aminotransferase in WD/CCl_4_-treated mice was only slightly higher than that of normal groups, suggesting a low degree of hepatitis (Figure S4). Taken together, these data suggest the WD/CCl_4_-treated mice after thirteen-week had been developing certain degree of fatty livers, with several characteristics of lean NAFLD patients, e.g., fatty liver and reduced weight.

In contrast, when EGCG (100 mg/kg) or PTM (10 mg/kg) was given daily to WD/CCl_4_-treated mice simultaneously, their fasting blood glucose decreased and the glucose tolerance increased, accompanying by a decrease of their body weights (Figure 2). Furthermore, the serum TG levels were also reduced, while the serum TC levels of EGCG and PTM-treated mice were significantly higher than those of WD/CCl_4_-treated mice (Figure 2c,d). The reason for this could be that the specific inhibition of EGCG or PTM of FASN reduced de novo fatty acid production, which promoted a compensation mechanism for the biosynthesis of sterol in mice. Histological analysis of liver tissues of EGCG-treated mice exhibited reduced accumulation of lipid droplets, while a lower dose of PTM (10 mg/kg) resulted in even significantly less lipid production in the livers of treated mice than the higher dose of PTM (50 mg/kg) (Figure 2g,h). These data suggest that both PTM and EGCG have protective effects against WD/CCl_4_ induced NAFLD formation. 

In an alternative protocol, PTM was given to WD/CCl_4_-treated mice after eight weeks, by oral administration (50 mg/kg) or IP injection (10 mg/kg), along with Lip/PTM and M/Lip-PTM (10 mg/kg), two liposomal formulations of PTM. The goal is to evaluate if these treatments may reverse certain pathological features induced by WD/CCl_4_. Although the body weight of mice treated by PTM (10 mg/kg, IP injection) decreased during the treatment, there was no significant change of the body weight of mice treated by both PTM nano-formulations (Figure 2). Although there were no significant changes of the levels of TC and TG in the plasma of treated mice, the level of TG in the livers of mice treated by M-Lip/PTM was reduced to the level of normal mice (Figure S2). This is also consistent with the significant reduced lipid accumulation in mouse liver tissues observed in HE staining and Oil red O staining, in which M/Lip-PTM and PTM (10 mg/kg, IP injection) exhibited lowest lipid accumulation. Intriguingly, PTM (50 mg/kg) showed almost no effects towards lipid accumulation in WD/CCl_4_ treated mice (Figure 2g,h). These data suggest that proper amount of PTM and the targeted delivery of PTM to mouse liver may be critical to achieve a superior protective effect against NAFLD formation.

### 3.2. PTM and PTM-Loaded Liposome Nanoparticles Attenuate Protein Abundance of FASN in Mouse Livers

**Figure 3:**
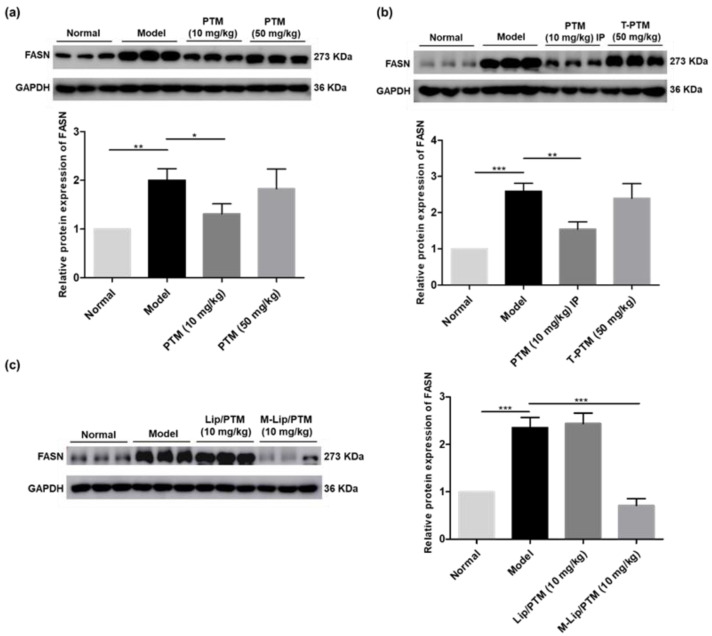
Effects of PTM and PTM-loaded liposome nanoparticles on FASN protein expression in mouse liver tissue. (**a**) Western blot analysis of FASN protein expression after 10 mg/kg and 50 mg/kg PTM oral treatment for 13 weeks. (**b**) Western blot analysis of FASN protein expression after 50 mg/kg PTM oral treatment and 10 mg/kg PTM intraperitoneal injection treatment for 5 weeks. (**c**) Western blot analysis of FASN protein expression after 10 mg/kg Lip/PTM and 10 mg/kg M-Lip/PTM intraperitoneal injection treatment for 5 weeks. Data are expressed as means ± SD, *n* = 3 mice per group. * *p* < 0.05, ** *p* < 0.01, *** *p* < 0.001.

In order to understand the pharmacological role of PTM and its liposomal nanoparticles in preventing the development of murine NAFLD induced by WD/CCl_4_**,** we further studied if PTM could inhibit the expression of FASN in the livers of treated mice. Using eDIO mice, Singh and co-workers showed that a four-day PTM treatment (100 mg/kg, p.o.) reduced ~40% of FASN protein in their livers [26]. Compared with the normal group, the protein expression of FASN was about two-fold higher in mice treated with WD/CCl_4_ for 13-weeks (Figure 3a). In contrast, PTM (10 mg/kg) significantly reduced the level of FASN. Interestingly, there was no significant change of FASN in liver tissues of mice treated by PTM (50 mg/kg), which is consistent with the previous histological results. Similarly, PTM (10 mg/kg, IP injection) was able to reduce the protein level of FASN significantly in model mice, while the higher dosage of PTM have no obvious effects towards FASN (Figure 3b). Encouragingly, M-Lip/PTM (10 mg/kg, IP injection) was able to reduce the protein level of FASN to that of normal mice, while Lip/PTM seemed to have no effects (Figure 3c). These results suggest that PTM may prevent the development of NAFLD effectively at a proper dose, i.e., 10 mg/kg. Notably, the liver-specific liposomal delivery of PTM containing mannose was capable to reduce FASN overexpression in mouse liver to that of normal mice for a shorter period.

### 3.3. PTM Treatment Alleviated FFAs-Induced Lipid Accumulation in HepG2 Cells

**Figure 4:**
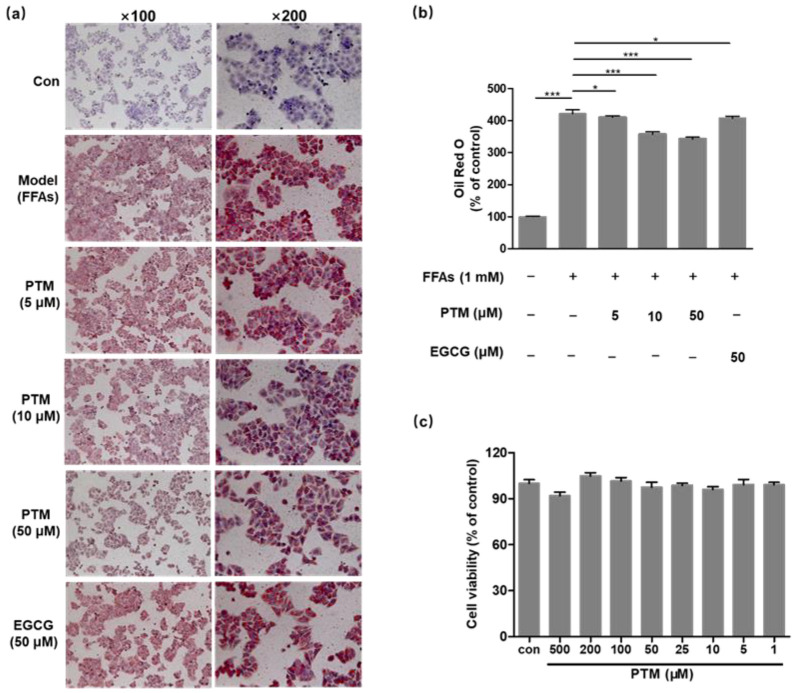
In vitro cytotoxicity and effect of PTM on lipid accumulation. (**a**) Effect of PTM on lipid accumulation in HepG2 cells. (**b**) Lipid content was extracted from Oil red O stained cells by isopropanol and quantified by spectrophotometric analysis at 510 nm. (**c**) Effects of PTM on HepG2 cell viability in response to different concentrations of PTM after incubation for 72 h. Data was visualized by GraphPad software. Error bars represent means ± SD. * *p* < 0.05, *** *p* < 0.001.

Various 2D and 3D human cell models have been developed to study the sophisticated pathology of NAFLD with primary human hepatocytes or hepatoma cell lines, including HepaRG, HepG2, and Huh 7 [30]. In order to form the steatotic cell phenotype, we treated HepG2 cells with the complex of BSA and oleic acid/palmitic acid at 0.4 and 0.2 mM with PTM (5, 10, 50 μM) or EGCG (50 μM) for 48 h, respectively, based on previous reports [30]. Oil red O staining was used to observe the accumulation of lipid droplets. Marked lipid accumulation was clearly observed in HepG2 cells treated with fatty acids, in comparison to the untreated cells. PTM significantly reduced the lipid droplet formation in the treated HepG2 cells in a dose-dependent manner, whereas EGCG also reduced the accumulation of lipid droplets (Figure 4a,b). In order to study if the inhibitory effect of PTM on lipid accumulation was caused by its cytotoxicity, cell viability tests of PTM were carried out using the MTT assay. PTM showed no cytotoxicity in the range of 5–500 μM against HepG2 cells, consistent with the previous report that PTM has no cytotoxicity towards mammalian cells (Figure 4c) [25].

### 3.4. PTM Affects the Levels of mRNA and Protein Expression of Lipid Metabolism in HepG2 Cells

**Figure 5:**
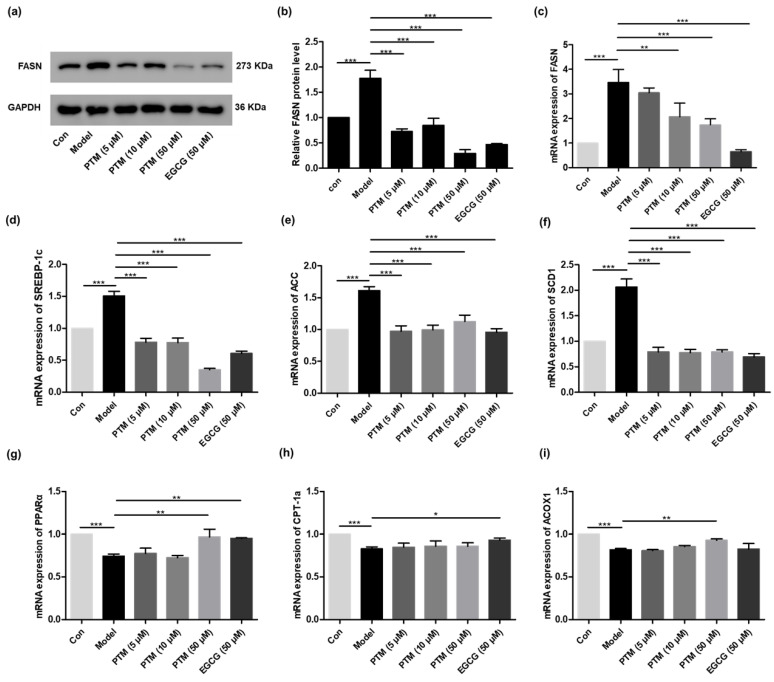
Effects of PTM on mRNA and protein abundance of target proteins involved in lipid metabolism in HepG2 cells. (**a**) Western blot analysis of the effects of PTM on the level of FASN, using EGCG as a control. (**b**) Protein blot densities were quantified by ImageJ Software; protein expression was normalized to GAPDH. (**c**)–(**i**). Quantitative RT-PCR analyses of the mRNA levels of FASN, SREBP-1c, ACC, SCD1, CPT-1a, PPARα, and ACOX1, relative to the internal control β-actin. Data were visualized by GraphPad software. Error bars represent means ± SD. * *p* < 0.05, ** *p* < 0.01, *** *p* < 0.001.

To explore the mode of action of PTM against fatty acid-induced steatosis in HepG2 cells, we first examined its effects towards the expression of *fasn* and its protein level, using EGCG as a control. Compared with the untreated HepG2 cells, supplementation of fatty acids increased cellular FASN, while PTM and EGCG treatment obviously attenuated the cellular production of FASN (Figure 5a,b). Quantitative real-time PCR revealed the increased mRNA level of FASN in fatty acid-treated HepG2 cells and the significant reduction of *fasn* expression upon PTM treatment in a dose-dependent manner, in comparison to untreated ones (Figure 5c). Notably, PTM (50 μM) could reduce the levels of both FASN protein and its mRNA in fatty acids-treated HepG2 cells lower than those of untreated HepG2 cells. This result is consistent with the potent inhibitory effect of PTM in rat primary hepatocytes, whereas PTM inhibited the biosynthesis of free fatty acids with an IC_50_ of 0.063 μM [24]. Since the de novo synthesis of fatty acids in HepG2 cells is catalyzed by FASN, we ascribed the difference of inhibitory effects of fatty acids and FASN to the different cell types and culture conditions with or without the supplementation of fatty acids.

Since FASN is a key node of cellular lipid metabolism, we next evaluated if FASN inhibition by PTM in HepG2 cells may have certain global effects towards lipid metabolism. EGCG (50 μM) was also used as a control. Therefore, quantitative RT-PCR was used to study the expression of key enzymes for cellular lipogenesis in fatty acid-treated HepG2 cells with or without PTM treatment, including sterol regulatory element binding transcription factor-1c (SREBP-1c), acetyl-CoA carboxylase (ACC), and stearoyl-CoA desaturase 1 (SCD1), as well as β-oxidation including carnitine palmitoyl transferase 1a (CPT-1a), peroxisome proliferator activated receptor α (PPARα), and acyl-CoA oxidase 1 (ACOX1) (Figure 5d–f). In our study, the mRNA levels of SREBP-1c, ACC, and SCD1 significantly increased in fatty acids-treated HepG2, compared to those in untreated ones. However, their expressions could be reduced to those of HepG2 cells without fatty acid supplementation upon PTM treatment. However, in fatty acids-induced HepG2 cells, the gene expression of PPARα, CPT-1a, and ACOX1 responsible for lipid oxidation significantly decreased, in comparison to uninduced ones. At the concentration of 50 μM, PTM and EGCG could enhance the expression of PPARα in fatty acids-induced HepG2 cells to that of uninduced ones. PTM (50 μM) slightly improved the expression of ACOX1, but not CPT-1a, which could be increased by EGCG. These data suggested that PTM is preferably peroxisomal *β*-oxidation rather than mitochondrial *β*-oxidation (Figure 5g–i). 

Fatty acid mitochondrial and peroxisomal β-oxidation is the central oxidative pathway for lipids, and CPT-1a is a significant rate-limiting enzyme for mitochondrial *β*-oxidation [34]. The entry of fatty acids into mitochondria relies on CPT-1a. Nevertheless, as mitochondria cannot oxidize very long chain fatty acids, they are metabolized via peroxisomal *β*-oxidation that is regulated by ACOX1. Taken together, PTM may attenuate fatty acids-induced lipid accumulation in HepG2 cells mainly through the downregulation of lipogenesis.

## 4. Discussion

NAFLD is the most prevalent chronic liver disease without effective pharmacological intervention in clinics. FASN is a promising target for NAFLD treatment, due to its rate-limiting capacity to control hepatic DNL. In the present study, we provide the first evidence that PTM and PTM-loaded liposomes could prevent the development of NAFLD in a murine model induced by WD/CCl_4_. Using a FFAs-induced HepG2 cell model, we showed that PTM could attenuate lipid accumulation through the down-regulation of FASN and several key proteins for lipogenesis, including SREBP-1c, ACC, and SCD1. This result suggests that the FASN inhibitor PTM may be used to intervene in the development of NAFLD. 

PTM has previously been discovered as a potent inhibitor against bacterial FabF, and it was latter re-discovered as a potent and highly selective inhibitor of mammalian FASN to be effective against type II diabetic animals. Considering the bidirectional relationship between NASH and type II diabetes, we envisioned that PTM might be effective against the development NAFLD by targeting FASN similarly. In FFAs-treated HepG2 cells, we showed that PTM alleviated the lipid droplet formation in a dose-dependent manner, mainly through down-regulating the expression of FASN, SREBP-1c, ACC, and SCD1. Importantly, PTM (5 μM) was able to reduce the expression of SREBP-1c, ACC, and SCD1 to that of uninduced HepG2 cells, suggesting that PTM-mediated FASN inhibition would have a global impact in lipid metabolism (Figure 5). In the WD/CCl_4_ murine model, the chronic treatment of PTM (10 mg/kg) for 13 weeks significantly reduced the protein level of FASN in the livers of treated mice, corroborating attenuated liver steatosis and reduced TG in their livers and plasma (Figure 2 and Figure S2). Further, the five-week treatment regime by M/Lip-PTM or PTM (10 mg/kg) could also reduce steatosis and the level of TG in the livers of treated mice, while FASN in mouse livers was also significantly reduced. 

However, higher PTM concentrations (50 mg/kg) exhibited no obvious effects against NAFLD, resulting in only slightly reduced FASN levels and liver steatosis. We assumed the presence of a certain compensatory mechanism upon stronger FASN inhibition. The liver-specific FASN knock-out (FASKOL) mice even developed hypoglycemia and fatty liver on a zero-fat diet, which was reasoned as the interference of PPARα activation, disruption of the transcription of genes critical for fatty acid oxidation, as well as interfered gluconeogenesis [35]. PTM (50 mg/kg) may cause accumulation of malonyl-CoA, which would inhibit CPT-1a and the entry of fatty acyl-CoA into mitochondria, resulting in suppressing *β*-oxidation [26]. The overall effect of significant FASN inhibition may be translated into the elevation of hepatic TG, despite the net consequence of FASN inhibition is to reduce the synthesis of TG. Intriguingly, there was no change of the mRNA level of CPT-1a in HepG2 cells treated by PTM (50 μM) (Figure 5h). We ascribed the difference to the dosage effects between the mouse model and HepG2 cells treated with oleic acid and palmitic acid.

Finally, the contribution of liver DNL varies dramatically in different species and disease states. For example, there are about 25% DNL in lean mice and 60% DNL in *db*/*db* mice, while DNL may contribute to 7–9% of plasma fatty acids in human and non-human primates, as well as 26% of them in NAFLD patients with hyperglycemia and hyperinsulinemia [26]. Thus, the effective dose of PTM needs to be further explored. Alternatively, manipulating the regulatory pathway of FASN under pathological conditions is a promising method for the treatment of NAFLD, such as the recent identified FASN-binding sorting nexin 8 [34,36]. The direct inhibition of FASN might trigger chronic on-target safety risks due to the reduced *β*-oxidation of fatty acids by compromising the activity of PPARα, since FASKOL mice showed lipid accumulation and hypoglycemia with a zero-fat diet or fasting. Considering the superior anti-NAFLD effects of a lower dosage of PTM (10 vs. 50 mg/kg) in the murine NAFLD model, the judicious selection of dosage of FASN inhibitors may be critical to achieve certain clinical benefits. It is encouraging that several recent clinical trials have demonstrated that FASN inhibitors can reduce hepatic DNL and steatosis in patients with NAFLD [17,21,23].

In summary, we have shown that PTM and PTM-loaded liposome nanoparticles could be used to reduce the development of NAFLD in a murine model through modulating DNL via FASN inhibition. Our study not only shows the potential of PTM against NAFLD, but highlights its multi-faceted applications against metabolic diseases and deadly pathogens.

## Data Availability

The original contributions presented in the study are included within the article/Supplementary Material, and further inquiries can be directed to the corresponding author.

## Supplementary Materials

The following are available online at https://www.mdpi.com/article/10.3390/biomedicines10010005/s1, Table S1. Primers used for real-time quantitative PCR; Figure S1. The body weights of mice treated by WD/CCl4; Figure S2. The hepatic total cholesterol and triglycerides of mice induced by WD/CCl4; Figure S3. The AUC of OGTT of treated mice by WD/CCl_4_; Figure S4. The plasma level of alanine aminotransferase of mice treated by WD/CCl_4_.

## References

[1] Younossi Z.M., Koenig A.B., Abdelatif D., Fazel Y., Henry L., Wymer M. (2016). Global Epidemiology of Nonalcoholic Fatty Liver Disease-Meta-Analytic Assessment of Prevalence, Incidence, and Outcomes. Hepatology.

[2] Fan J.G., Kim S.U., Wong V.W. (2017). New Trends on Obesity and NAFLD in Asia. J. Hepatol..

[3] Zhou J., Zhou F., Wang W., Zhang X.J., Ji Y.X., Zhang P., She Z.G., Zhu L., Cai J., Li H. (2020). Epidemiological Features of NAFLD from 1999 to 2018 in China. Hepatology.

[4] Golabi P., Paik J.M., Arshad T., Younossi Y., Mishra A., Younossi Z.M. (2020). Mortality of NAFLD According to the Body Composition and Presence of Metabolic Abnormalities. Hepatol. Commun..

[5] Ye Q., Zou B., Yeo Y.H., Li J., Huang D.Q., Wu Y., Yang H., Liu C., Kam L.Y., Tan X.X.E. (2020). Global Prevalence, Incidence, and Outcomes of Non-Obese or Lean Non-Alcoholic Fatty Liver Disease: A Systematic Review and Meta-Analysis. Lancet Gastroenterol. Hepatol..

[6] Khan R.S., Newsome P.N. (2016). Non-Alcoholic Fatty Liver Disease and Liver Transplantation. Metabolism.

[7] Jayawardena R., Sooriyaarachchi P., Chourdakis M., Jeewandara C., Ranasinghe P. (2020). Enhancing Immunity in Viral Infections, with Special Emphasis on COVID-19: A Review. Diabetes Metab. Syndr..

[8] Meijnikman A.S., Bruin S., Groen A.K., Nieuwdorp M., Herrema H. (2021). Increased Expression of Key SARS-COV-2 Entry Points in Multiple Tissues in Individuals with NAFLD. J. Hepatol..

[9] Smith G.I., Shankaran M., Yoshino M., Schweitzer G.G., Chondronikola M., Beals J.W., Okunade A.L., Patterson B.W., Nyangau E., Field T. (2020). Insulin Resistance Drives Hepatic De Novo Lipogenesis in Nonalcoholic Fatty Liver Disease. J. Clin. Invest..

[10] Chiu S., Mulligan K., Schwarz J.M. (2018). Dietary Carbohydrates and Fatty Liver Disease: De Novo Lipogenesis. Curr. Opin. Clin. Nutr. Metab. Care.

[11] Schwarz J.M., Clearfield M., Mulligan K. (2017). Conversion of Sugar to Fat: Is Hepatic De Novo Lipogenesis Leading to Metabolic Syndrome and Associated Chronic Diseases?. J. Am. Osteopath. Assoc..

[12] Alkhouri N., Lawitz E., Noureddin M., DeFronzo R., Shulman G.I. (2020). Gs-0976 (Firsocostat): An Investigational Liver-Directed Acetyl-CoA Carboxylase (ACC) Inhibitor for the Treatment of Non-Alcoholic Steatohepatitis (NASH). Expert Opin. Investig. Drugs.

[13] Bates J., Vijayakumar A., Ghoshal S., Marchand B., Yi S., Kornyeyev D., Zagorska A., Hollenback D., Walker K., Liu K. (2020). Acetyl-CoA Carboxylase Inhibition Disrupts Metabolic Reprogramming During Hepatic Stellate Cell Activation. J. Hepatol..

[14] Harriman G., Greenwood J., Bhat S., Huang X., Wang R., Paul D., Tong L., Saha A.K., Westlin W.F., Kapeller R. (2016). Acetyl-CoA Carboxylase Inhibition by ND-630 Reduces Hepatic Steatosis, Improves Insulin Sensitivity, and Modulates Dyslipidemia in Rats. Proc. Natl. Acad. Sci. USA.

[15] Kim C.W., Addy C., Kusunoki J., Anderson N.N., Deja S., Fu X., Burgess S.C., Li C., Ruddy M., Chakravarthy M. (2017). Acetyl CoA Carboxylase Inhibition Reduces Hepatic Steatosis but Elevates Plasma Triglycerides in Mice and Humans: A Bedside to Bench Investigation. Cell Metab..

[16] Lawitz E.J., Coste A., Poordad F., Alkhouri N., Loo N., McColgan B.J., Tarrant J.M., Nguyen T., Han L., Chung C. (2018). Acetyl-CoA Carboxylase Inhibitor GS-0976 for 12 Weeks Reduces Hepatic De Novo Lipogenesis and Steatosis in Patients with Nonalcoholic Steatohepatitis. Clin. Gastroenterol. Hepatol..

[17] Loomba R., Kayali Z., Noureddin M., Ruane P., Lawitz E.J., Bennett M., Wang L., Harting E., Tarrant J.M., McColgan B.J. (2018). GS-0976 Reduces Hepatic Steatosis and Fibrosis Markers in Patients with Nonalcoholic Fatty Liver Disease. Gastroenterology.

[18] Wu X., Huang T. (2020). Recent Development in Acetyl-CoA Carboxylase Inhibitors and Their Potential as Novel Drugs. Future Med. Chem..

[19] Goedeke L., Bates J., Vatner D.F., Perry R.J., Wang T., Ramirez R., Li L., Ellis M.W., Zhang D., Wong K.E. (2018). Acetyl-CoA Carboxylase Inhibition Reverses NAFLD and Hepatic Insulin Resistance but Promotes Hypertriglyceridemia in Rodents. Hepatology.

[20] Dorn C., Riener M.O., Kirovski G., Saugspier M., Steib K., Weiss T.S., Gabele E., Kristiansen G., Hartmann A., Hellerbrand C. (2010). Expression of Fatty Acid Synthase in Nonalcoholic Fatty Liver Disease. Int. J. Clin. Exp. Pathol..

[21] Beysen C., Schroeder P., Wu E., Brevard J., Ribadeneira M., Lu W., Dole K., O’Reilly T., Morrow L., Hompesch M. (2021). Inhibition of Fatty Acid Synthase with FT-4101 Safely Reduces Hepatic De Novo Lipogenesis and Steatosis in Obese Subjects with Non-Alcoholic Fatty Liver Disease: Results from Two Early-Phase Randomized Trials. Diabetes Obes. Metab..

[22] Rohrbach T.D., Asgharpour A., Maczis M.A., Montefusco D., Cowart L.A., Bedossa P., Sanyal A.J., Spiegel S. (2019). FTY720/Fingolimod Decreases Hepatic Steatosis and Expression of Fatty Acid Synthase in Diet-Induced Nonalcoholic Fatty Liver Disease in Mice. J. Lipid Res..

[23] Syed-Abdul M.M., Parks E.J., Gaballah A.H., Bingham K., Hammoud G.M., Kemble G., Buckley D., McCulloch W., Manrique-Acevedo C. (2020). Fatty Acid Synthase Inhibitor TVB-2640 Reduces Hepatic De Novo Lipogenesis in Males with Metabolic Abnormalities. Hepatology.

[24] Wu M., Singh S.B., Wang J., Chung C.C., Salituro G., Karanam B.V., Lee S.H., Powles M., Ellsworth K.P., Lassman M.E. (2011). Antidiabetic and Antisteatotic Effects of the Selective Fatty Acid Synthase (FAS) Inhibitor Platensimycin in Mouse Models of Diabetes. Proc. Natl. Acad. Sci. USA.

[25] Wang J., Soisson S.M., Young K., Shoop W., Kodali S., Galgoci A., Painter R., Parthasarathy G., Tang Y.S., Cummings R. (2006). Platensimycin Is a Selective FabF Inhibitor with Potent Antibiotic Properties. Nature.

[26] Singh S.B., Kang L., Nawrocki A.R., Zhou D., Wu M., Previs S., Miller C., Liu H., Hines C.D., Madeira M. (2016). The Fatty Acid Synthase Inhibitor Platensimycin Improves Insulin Resistance without Inducing Liver Steatosis in Mice and Monkeys. PLoS ONE.

[27] Muzica C.M., Sfarti C., Trifan A., Zenovia S., Cuciureanu T., Nastasa R., Huiban L., Cojocariu C., Singeap A.M., Girleanu I. (2020). Nonalcoholic Fatty Liver Disease and Type 2 Diabetes Mellitus: A Bidirectional Relationship. Can. J. Gastroenterol. Hepatol..

[28] Shi J., Pan J., Liu L., Yang D., Lu S., Zhu X., Shen B., Duan Y., Huang Y. (2016). Titer Improvement and Pilot-Scale Production of Platensimycin from Streptomyces Platensis SB12026. J. Ind. Microbiol. Biotechnol..

[29] Wang Z., Liu X., Peng Y., Su M., Zhu S., Pan J., Shen B., Duan Y., Huang Y. (2020). Platensimycin-Encapsulated Liposomes or Micelles as Biosafe Nanoantibiotics Exhibited Strong Antibacterial Activities against Methicillin-Resistant Staphylococcus Aureus Infection in Mice. Mol. Pharm..

[30] Soret P.A., Magusto J., Housset C., Gautheron J. (2020). In Vitro and in Vivo Models of Non-Alcoholic Fatty Liver Disease: A Critical Appraisal. J. Clin. Med..

[31] Tsuchida T., Lee Y.A., Fujiwara N., Ybanez M., Allen B., Martins S., Fiel M.I., Goossens N., Chou H.I., Hoshida Y. (2018). A Simple Diet- and Chemical-Induced Murine Nash Model with Rapid Progression of Steatohepatitis, Fibrosis and Liver Cancer. J. Hepatol..

[32] Chen C., Liu Q., Liu L., Hu Y.Y., Feng Q. (2018). Potential Biological Effects of (-)-Epigallocatechin-3-Gallate on the Treatment of Nonalcoholic Fatty Liver Disease. Mol. Nutr. Food Res..

[33] Dong L.B., Rudolf J.D., Lin L., Ruiz C., Cameron M.D., Shen B. (2017). In Vivo Instability of Platensimycin and Platencin: Synthesis and Biological Evaluation of Urea- and Carbamate-Platensimycin. Bioorg. Med. Chem..

[34] Veeramani C., Alsaif M.A., Al-Numair K.S. (2018). Lavatera Critica Controls Systemic Insulin Resistance by Ameliorating Adipose Tissue Inflammation and Oxidative Stress Using Bioactive Compounds Identified by GC-MS. Biomed. Pharmacother..

[35] Chakravarthy M.V., Pan Z., Zhu Y., Tordjman K., Schneider J.G., Coleman T., Turk J., Semenkovich C.F. (2005). "New" Hepatic Fat Activates Pparalpha to Maintain Glucose, Lipid, and Cholesterol Homeostasis. Cell Metab..

[36] Hu Y., He W., Huang Y., Xiang H., Guo J., Che Y., Cheng X., Hu F., Hu M., Ma T. (2021). Fatty Acid Synthase-Suppressor Screening Identifies Sorting Nexin 8 as a Therapeutic Target for NAFLD. Hepatology.

