# Supplementary material for: Fatty Acid Synthase Inhibitor Platensimycin Intervenes the Development of Nonalcoholic Fatty Liver Disease in a Mouse Model
Source: Biomedicines. 2021 Dec 21;10(1):5. doi: 10.3390/biomedicines10010005 (PMC8773228; doi:10.3390/biomedicines10010005)
Supplement: Supplementary file 1 [file biomedicines-10-00005-s001.zip › biomedicines-1459268-supplementary.pdf]

Supporting Information

Fatty Acid Synthase Inhibitor Platensimycin Intervenes the Development of Nonalcoholic-Fatty Liver Disease in a Mouse Model

Meng Su[1†], Danfeng Cao[1†], Zhe Wang[1], Yanwen Duan[1,2,3*], and Yong Huang[1,3*]

[1]Xiangya International Academy of Translational Medicine at Central South University, Changsha, Hunan 410013, China;
[2]Hunan Engineering Research Center of Combinatorial Biosynthesis and Natural Product Drug Discovery, [3]National Engineering Research Center of Combinatorial Biosynthesis for Drug Discovery, Changsha, Hunan 410011, China;

[†]These authors contributed equally to this work.

ADDRESS CORRESPONDENCE AND REPRINT REQUESTS TO:
Yong Huang; or Yanwen Duan
Xiangya International Academy of Translational Medicine college,
Central South University,
Tongzipo Road, Changsha, Hunan 410013, China;
E-mail: jonghuang@csu.edu.cn (YH) or ywduan66@csu.edu.cn (YD)
Tel.: 0731-82650539

Table of contents

Table S1. Primers used for quantitative real-time PCR 3

Figure S1. The body weights of mice treated by WD/CCl_4. 4

Figure S2. The hepatic total cholesterol and triglycerides of mice induced by WD/CCl_4...5

Figure S3. The AUC of OGTT of treated mice by WD/CCl_4. 6

Figure S4. The plasma level of alanine aminotransferase of mice treated by WD/CCl_4. 7

Table S1. Primers used for quantitative real-time PCR.

Gene	Sequence (5′—3′)
FASN	Sense: AAGGACCTGTCTAGGTTTGATGC
	Antisense: TGGCTTCATAGGTGACTTCCA
ACC	Sense: ATGTCTGGCTTGCACCTAGTA
	Antisense: CCCCAAAGCGAGTAACAAATTCT
SREBP-1c	Sense: ACAGTGACTTCCCTGGCCTAT
	Antisense: GCATGGACGGGTACATCTTCAA
SCD	Sense: TCTAGCTCCTATACCACCACCA
	Antisense: TCGTCTCCAACTTATCTCCTCC
CPT-1a	Sense: ATCAATCGGACTCTGGAAACGG
	Antisense: TCAGGGAGTAGCGCATGGT
PPARα	Sense: TTCGCAATCCATCGGCGAG
	Antisense: CCACAGGATAAGTCACCGAGG
ACOX1	Sense: ACTCGCAGCCAGCGTTATG
	Antisense: AGGGTCAGCGATGCCAAAC
β-actin	Sense: CATGTACGTTGCTATCCAGGC
	Antisense: CTCCTTAATGTCACGCACGAT

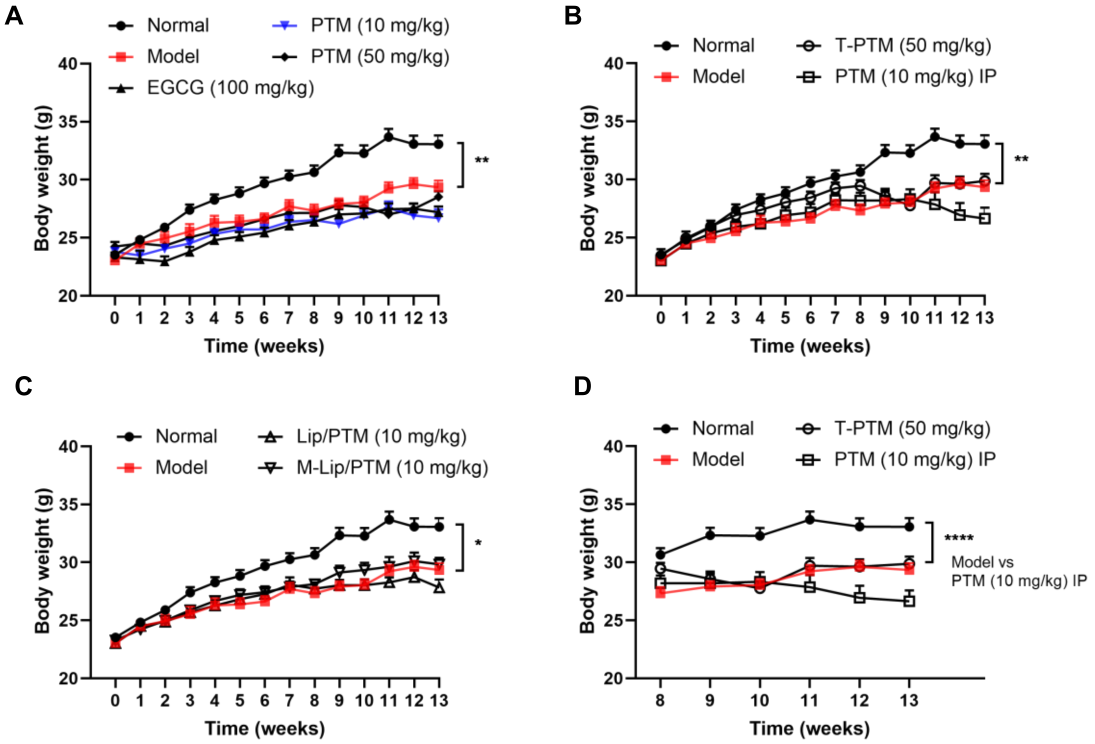

Figure S1. The body weights of mice treated by WD/CCl_4. (**A** – **C**) The body weights of mice treated by western diet and CCl_4. (**D**). The body weight change of 10 mg/kg PTM IP group from week 8th to week 13th. *$P < 0.05$, **$P < 0.01$, ****$P < 0.0001$.

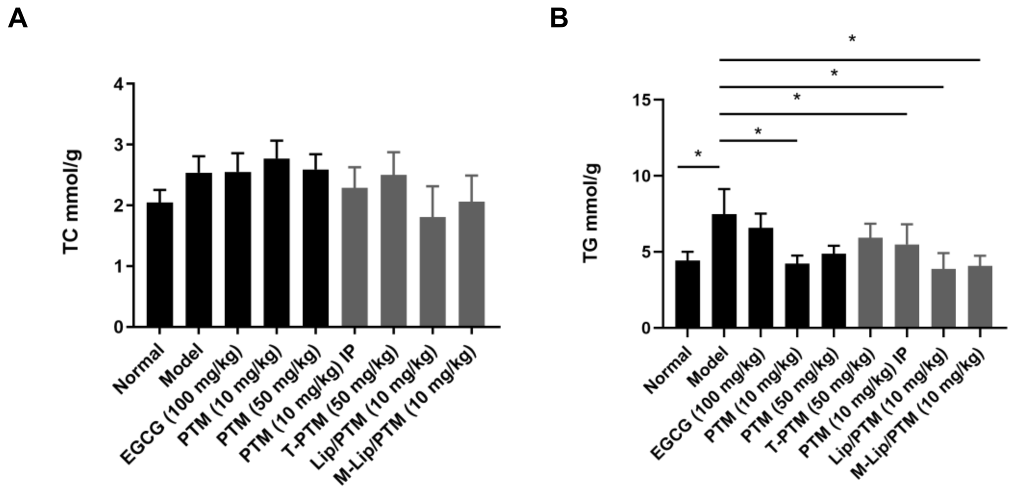

Figure S2. The hepatic total cholesterol and triglycerides of mice treated by WD/CCl_4. (**A**) The total cholesterol. (**B**) The total triglycerides. **P* < 0.05.

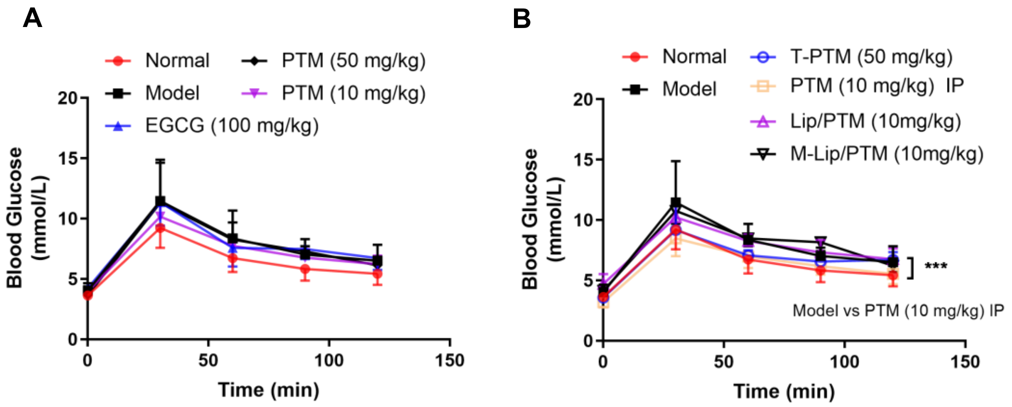

Fig S3. The AUC of OGTT of treated mice by WD/CCl_4. (**A**) 100 mg/kg EGCG, 10 mg/kg PTM and 50 mg/kg PTM: oral treatment for 13 weeks. (**B**) 50 mg/kg PTM oral treatment for 5 weeks; 10 mg/kg PTM, 10 mg/kg Lip/PTM, 10 mg/kg M-Lip/PTM: intraperitoneal injection for five weeks.

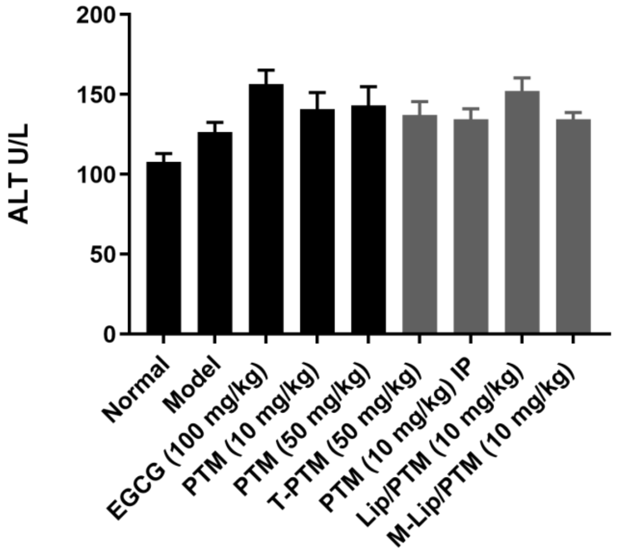

Figure S4. The plasma level of alanine aminotransferase of mice treated by WD/CCl_4.
